# The mouse Gene Expression Database (GXD): 2026 update

**DOI:** 10.1093/nar/gkaf1236

**Published:** 2025-11-20

**Authors:** Constance M Smith, Terry F Hayamizu, Jacqueline H Finger, Ingeborg J McCright, Jingxia Xu, Jeffrey Campbell, Lori E Corbani, Jake Emerson, Pete J Frost, Hongping Liang, Joel E Richardson, Richard M Baldarelli, Martin Ringwald

**Affiliations:** The Jackson Laboratory, Bar Harbor, ME 04609, United States; The Jackson Laboratory, Bar Harbor, ME 04609, United States; The Jackson Laboratory, Bar Harbor, ME 04609, United States; The Jackson Laboratory, Bar Harbor, ME 04609, United States; The Jackson Laboratory, Bar Harbor, ME 04609, United States; The Jackson Laboratory, Bar Harbor, ME 04609, United States; The Jackson Laboratory, Bar Harbor, ME 04609, United States; The Jackson Laboratory, Bar Harbor, ME 04609, United States; The Jackson Laboratory, Bar Harbor, ME 04609, United States; The Jackson Laboratory for Genomic Medicine, Farmington, CT 06032, United States; The Jackson Laboratory, Bar Harbor, ME 04609, United States; The Jackson Laboratory, Bar Harbor, ME 04609, United States; The Jackson Laboratory, Bar Harbor, ME 04609, United States

## Abstract

The Gene Expression Database (GXD; https://www.informatics.jax.org/expression.shtml) is an extensive, well-curated community resource that provides detailed information about gene expression patterns in mouse strains and mutants, with a particular emphasis on development. For over 25 years, GXD has systematically curated the scientific literature and collaborated with large-scale expression projects to compile and integrate detailed expression data from multiple assay types, including RNA *in situ* hybridization, immunohistochemistry, *in situ* reporter (knock-in), RT-PCR, northern blot, and western blot experiments. In recent years, GXD has expanded its scope to include bulk RNA-Seq data, imported from the EMBL-EBI Expression Atlas. Since our last report in 2021, we continued to add data to GXD on a daily basis, and we implemented new search and display features. These include: (i) searches for qualitative differential gene expression and expression profiles that include both classical types of expression data and RNA-Seq data; (ii) browsing, searching, and filtering for cell-type-specific gene expression information; and (iii) an enhancement of our RNA-Seq and microarray experiment metadata index and search to allow users to quickly and reliably find bulk, single-cell, or spatial RNA-Seq data in the public repositories.

## Graphical Abstract

**Graphical Abstract:**
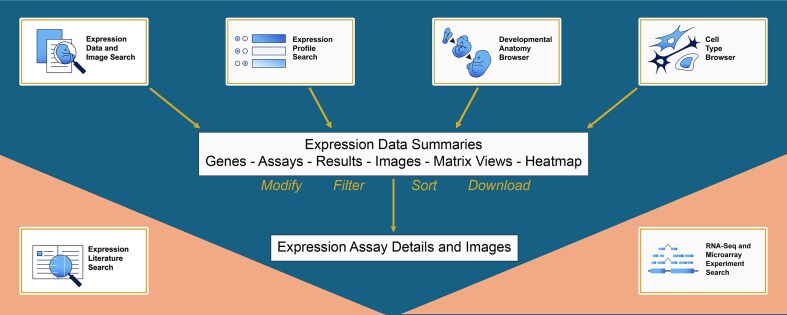

## Introduction

The Gene Expression Database (GXD; https://www.informatics.jax.org/expression.shtml) is a vital resource for researchers and others aiming to advance our understanding of the molecular basis of human development and disease. Developmental gene expression information from wild-type and mutant mice provides crucial insights into the molecular mechanisms of development, differentiation, and disease. Using a variety of expression assay types, scientists determine the transcripts and proteins generated from individual mouse genes, determine the spatial and temporal patterns of their expression, and examine how their expression patterns vary in different mouse strains and mutants. GXD annotates and integrates these complex data and makes them readily accessible to many types of database searches. Since its inception, GXD has collected RNA and protein expression information from what are now considered classical assay types—RNA *in situ* hybridization, immunohistochemistry, *in situ* reporter (knock-in), RT-PCR, northern blot, and western blot experiments [1–3]. These data are acquired from thousands of publications and through collaboration with projects doing large-scale expression screens [4–8]. More recently, GXD has expanded its scope to include bulk RNA-Seq data [9]. These data are imported from EMBL-EBI’s Expression Atlas and then further processed and annotated, resulting in seamless integration with GXD’s classical data. As an integral component of the larger Mouse Genome Informatics (MGI) resource [10, 11], GXD combines its expression data with genetic, functional, phenotypic, and disease-oriented data. This enables unique and powerful biologically and biomedically relevant searching. GXD and its user interfaces have been described previously [12–15]. Here, we focus on progress made since our last Nucleic Acids Research Database issue report [9].

## Data content and progress in data acquisition

### Comprehensive literature survey

GXD maintains an index of references that examine endogenous gene expression during mouse development. It can be accessed using the Gene Expression Literature Query Form (https://www.informatics.jax.org/gxdlit). To populate this index GXD curators systematically review journals to find all relevant studies that use classical *in situ* and blot assay types. They then annotate the genes and ages analyzed and the expression assay types used in these papers. Annotations are based on the entire publication, including supplementary data, and utilize official gene nomenclature to ensure precise gene identification. This information is then combined with bibliographic information from PubMed. GXD’s literature index is up-to-date and currently includes records for *>*34 000 references and data on >17 000 genes. This resource provides researchers with an effective tool for finding publications with specific gene expression data and helps GXD curators to prioritize papers for detailed expression annotation.

### Detailed annotation of classical types of expression data

GXD is a repository of detailed gene expression results derived from curation of publications in the literature index and large-scale expression projects. Using descriptions provided by the authors, the GXD curators make extensive use of controlled vocabularies and ontologies to annotate when and where genes are expressed. This data standardization facilitates its integration, enabling GXD’s powerful search capabilities.

**Figure 1.**
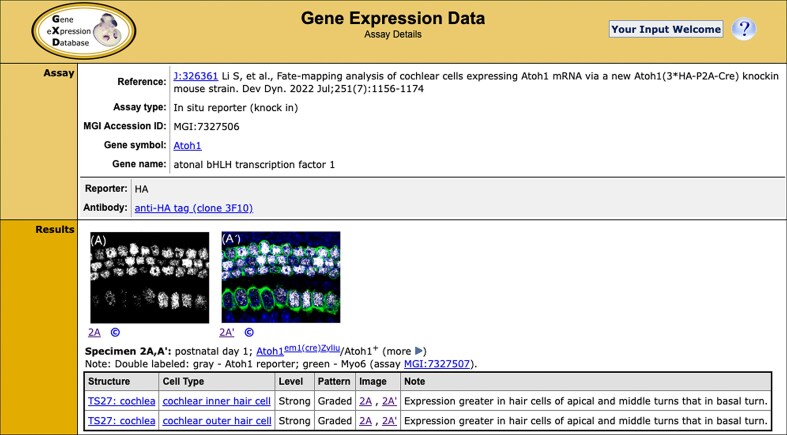
Detailed expression record. The Assay section at the top of the page displays the citation information, gene analyzed, and experiment information, such as the assay type, probe (or antibody) used, and visualization method. To supply additional context, there are links to pages with additional gene, reference, and probe details. Information about the expression patterns observed and specimen details can be found in the Results section. The expression pattern is described using terms from the Developmental Anatomy and Cell Ontologies. The level and pattern of expression is as reported by the authors. Image panes accompany the result annotations when permitted by the publisher; the full figure can be viewed by clicking on either the image or image link. Specimen details include the age, genetic background, and specimen preparation methods. Some specimen details are displayed by default; all details can be viewed by using the more ▸ toggle. Please note this figure only displays a portion of the expression record; the entire record can be viewed at https://www.informatics.jax.org/assay/MGI:7327506.

Expression patterns in GXD are annotated using the Mouse Developmental Anatomy Ontology (https://www.informatics.jax.org/vocab/gxd/anatomy), a comprehensive and hierarchically structured ontology [16, 17]. The hierarchical structure allows for the integration of expression results from assays with differing spatial resolution and allows expression searches by an anatomical term to include all its subterms. Since our last report, we have begun to use the Cell Ontology (CL) [18] as an additional standardized descriptor of expression patterns. Reports of expression in a particular cell type and tissue are now annotated in a modular way using terms from both ontologies. An example of such annotations, from an *in situ* reporter (knock-in) expression data record, is shown in Fig. 1.

In addition to standardized descriptions of expression patterns, detailed expression records also include the gene analyzed, assay type, age and genetic background of the specimens, and information about the probes and experimental conditions used. These data are recorded using standard mouse nomenclature for genes, strains, and alleles, and controlled vocabularies. In addition, when we have permission to display them, images of the data accompany the annotations. As of August 2025, GXD contains detailed classical expression data for >16 200 genes, including data from ∼8700 mouse mutants. Most of these data (82%) are from *in situ* hybridization, immunohistochemistry, and *in situ* reporter (knock-in) experiments. GXD now holds >2 million expression result annotations for classical types of expression data, covering 18 000 unique stage-specific anatomical structures, with ∼500 000 accompanying images.

### RNA-Seq expression data

GXD also integrates bulk RNA-Seq data examining endogenous gene expression in mouse strains and mutants. These data are imported from the Expression Atlas at the EMBL-EBI [19]. The Expression Atlas identifies high-quality bulk RNA-Seq expression data sets and uses a standardized processing pipeline to generate TPM values that are uniform and comparable across studies. To seamlessly integrate these data into GXD, we perform additional processing on the TPM files provided by the Expression Atlas. Specifically, we compute averaged quantile normalized TPM values per gene per biological replicate set. Then, using the Expression Atlas thresholds as a guide, we assign these values to expression bins of high, medium, low, and below cutoff (i.e. not detected). This binning enables us to assign a detected/not detected value to these data, as is done for all the other expression data in GXD. Additionally, we annotate the metadata for each biological replicate set (e.g. anatomical structure, developmental stage, genetic background, etc.) using the same controlled vocabularies and ontologies we use for all other samples and specimens in GXD. These steps enable the full integration of these data into GXD. A detailed description of this process is provided by Baldarelli *et al.* [9]. To date, GXD has incorporated expression data for a selected set of 96 RNA-Seq experiments. These include 2127 samples, representing 178 distinct anatomical structures and 160 different genetic backgrounds. Each experiment’s TPM file provides comprehensive genome coverage that spans the entire Ensembl transcriptome of nearly 52 000 genome features, including protein-coding and non-coding RNA genes. Comprehensive reports for each RNA-Seq experiment, including GXD-curated sample metadata and GXD-computed TPM values per gene, are also available for download at https://www.informatics.jax.org/downloads/reports/index.html#expression.

We also plan to incorporate single-cell RNA-Seq data from the EMBL-EBI’s Single-Cell Expression Atlas [19] into GXD by aggregating the data at the cell type (pseudo-bulk) level, i.e. by combining, for each dataset, the counts per gene from individual cells of reported cell clusters (per tissue). Pseudo-bulking has been used as a method to mediate some of the technical variation and sparsity of molecule detection inherent in scRNA-Seq data [20]. Further, this representation will be similar to the representation of bulk RNA-Seq data currently in GXD. Therefore, the single-cell RNA-Seq data will be well integrated with all the other expression data in GXD and accessible through most of the search utilities that GXD provides.

### Index of publicly available RNA-Seq and microarray experiments

GXD has developed an RNA-Seq and Microarray Experiment Search tool (https://www.informatics.jax.org/gxd/htexp_index) that allows users to efficiently locate experiments that study endogenous gene expression in wild-type and mutant mice in the major public repositories. These repositories, Gene Expression Omnibus (GEO) [21] and ArrayExpress [22], do not require that the metadata for data submissions be standardized. This results in widespread term heterogeneity, making it difficult to reliably find experiments of interest. GXD has developed a curated, searchable metadata index of RNA-Seq and microarray experiments that are consistent with GXD’s scope, i.e. those that examine endogenous gene expression in tissues from wild-type and mutant mice (both pre- and postnatal stages), as well as comparative studies between mouse and other species. GXD imports the experiment accession ID, title, summary, and raw sample data from the repository. Then, using controlled vocabularies and ontologies, GXD curators create standardized annotations for the anatomical structure, developmental stage, mutated gene, strain, and sex of the samples, and the study type and key parameters of each experiment. The RNA-Seq and Microarray Experiment Search enables interrogation of the indexed metadata in combination with free-text searching of experiment titles and descriptions, allowing for quick and reliable searches [23].

The high-throughput metadata index is comprised of experiments deposited in both GEO and ArrayExpress. Initially, we used ArrayExpress as our sole repository source because it imported all experiments stored at GEO, thus ensuring our download would include information from both repositories. ArrayExpress has since stopped its importation of experiments from GEO. Therefore, while we continue to download experiments from ArrayExpress, since our last report we have developed a new load to import experiments and their raw sample data directly from GEO, thus ensuring our index still encompasses experiments from both repositories. Additionally, to manage the surge of RNA-Seq experiments, we developed a machine learning classifier to help in identifying experiments relevant to GXD and incorporated it into this load. Leveraging the Random Forest algorithm [24], the classifier analyzes experiment titles, descriptions, and raw sample metadata. It achieves a negative predictive value of 97%, effectively filtering out non-relevant experiments, which allows GXD curators to focus their efforts on evaluating the experiments most likely to be suitable for GXD.

Bulk, single-cell, and spatial RNA-Seq experiments provide different types of expression information. Although these differences are of interest to researchers, they are not searchable at GEO. Since our last report, we have addressed this by annotating all RNA-Seq samples in our index to one of these three methods. We chose to annotate the RNA-Seq type at the sample, rather than experiment, level because individual datasets may contain a mix of RNA-Seq methods. These annotations allow users to either specify the method of interest in the initial search or, alternatively, refine their search results using a new Method filter found on the search summary. Currently, there are 7381 RNA-Seq experiments in the metadata index; 5454 are bulk studies, 1852 single-cell, and 139 spatial studies. In total there are ∼10 500 experiments in the index containing >220 000 annotated samples. There are > 4300 baseline studies using a variety of wild-type strains and ∼6200 wild-type versus mutant studies, using >5600 mutant alleles.

## Enhancements to the user interface

### New Expression Profile Search

**Figure 2.**
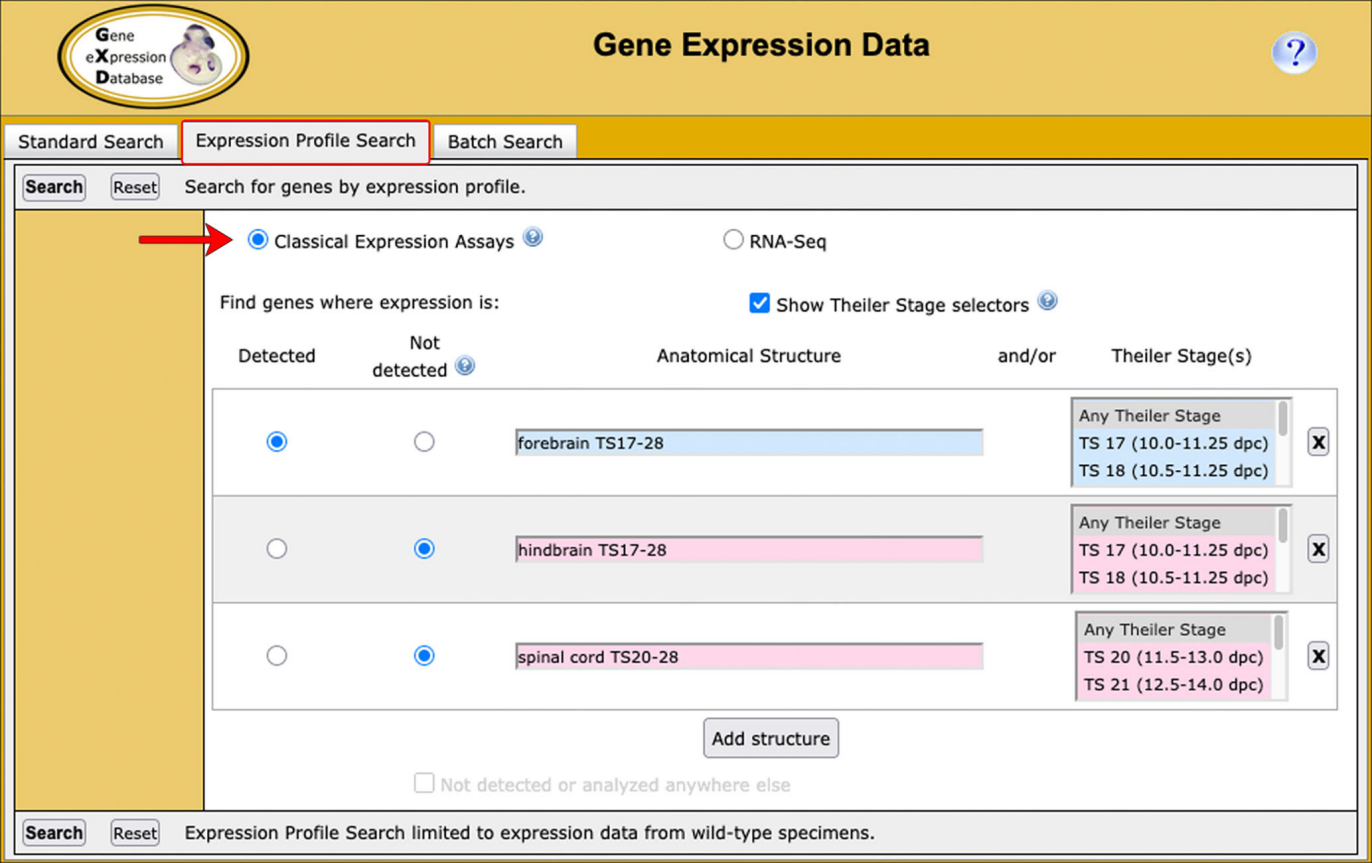
Expression Profile Search. To begin an Expression Profile Search, users first choose (arrow) which type of data they wish to interrogate: Classical Expression Assays or RNA-Seq data. The next step is to specify whether they wish gene expression to be detected (blue background) or not detected (red background) within the anatomical structure. Typing in the structure field activates an autocomplete list displaying matching anatomical structures and synonyms. Users must choose from this list. To specify developmental stages, users can check the “Show Theiler stage selectors” box. These selectors are hidden by default but are shown in the figure. This feature allows users to limit the search to specific stage(s) for the chosen anatomical structure or to search by stage(s) alone, without specifying an anatomical structure. Both the anatomical term and developmental stage lists are limited to terms or stages for which GXD has the selected type of expression data. More detailed instructions on how to use the Expression Profile Search are provided at https://www.informatics.jax.org/faq/GXD_exp_profile.shtml.

The GXD Home Page provides a comprehensive suite of search tools for accessing GXD’s detailed expression data (https://www.informatics.jax.org/expression.shtml). A recent addition to this toolkit is the GXD Expression Profile Search (https://www.informatics.jax.org/gxd/profile; Fig. 2). It enables users to identify genes based on their expression patterns, allowing them to find genes expressed in certain anatomical structures and/or developmental stages while absent in others. Originally launched in November 2022, the Expression Profile Search was limited to interrogations of classical expression data, i.e. *in situ* and blot data [11]. We have now redesigned the indices and code powering the profile searches, resulting in significantly improved search speeds. This has allowed expansion of the profile search to include RNA-Seq data. In addition, we have made this utility more versatile. Users have always been able to define expression profiles by selecting up to 10 anatomical structures and specifying whether expression is present or absent in these structures. Now, they can also specify the developmental (Theiler) stage(s) of interest. This allows users to search by developmental stage alone or combine anatomical structures with developmental stages. The addition of this functionality to the profile search allowed us to retire the Differential Expression search (released in 2018) as all its utilities are now available in the expanded, redesigned Expression Profile search.

**Figure 3.**
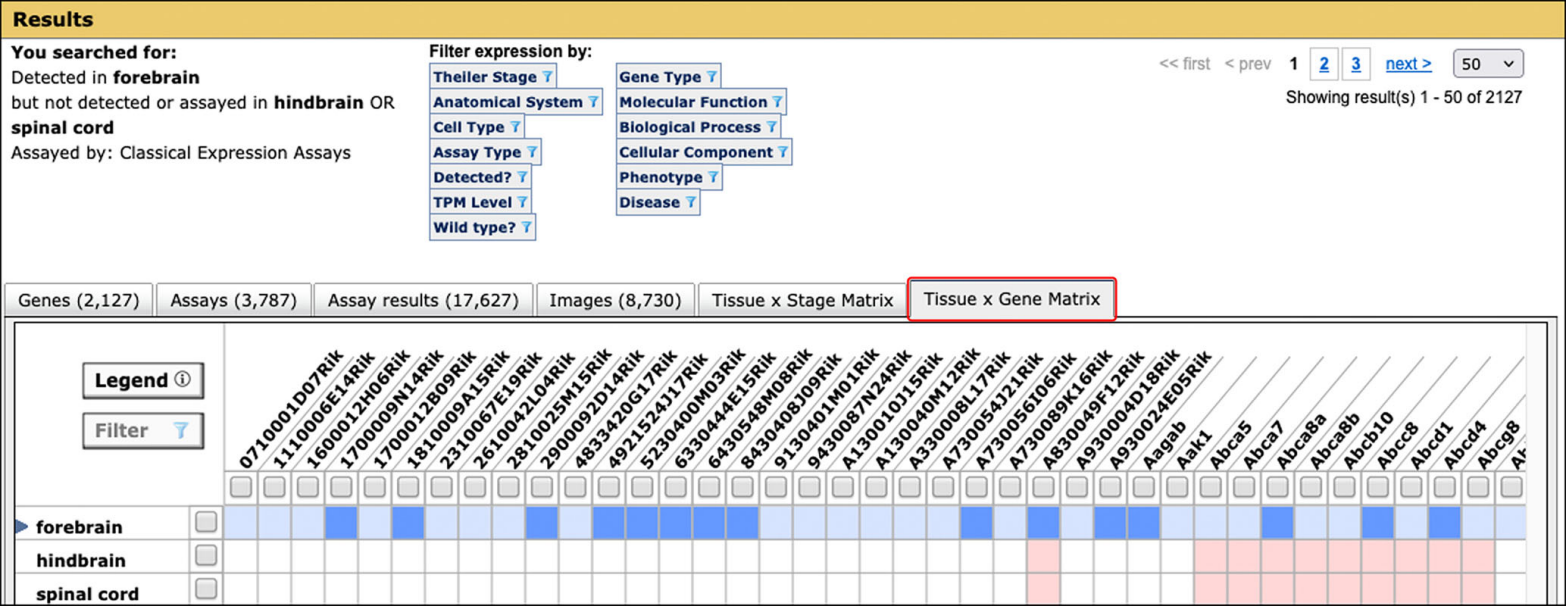
Tissue × Gene Matrix tab of the Gene Expression Search Summary. Searches for expression data return summaries with multiple tabbed views, allowing users to review the returned data at their preferred level of detail. When using the Classical Expression Assays mode of the Expression Profile Search, the default view is the Tissue × Gene Matrix (boxed in red). Columns in this matrix display the genes, and rows correspond to tissues matching the search criteria. Blue cells indicate expression was detected, red indicates that expression was not detected, and white that there are no annotations for the gene in this tissue. Colors get progressively darker when there are more supporting annotations. Users can expand or collapse the anatomy hierarchy by clicking the ▸toggle. Clicking on a cell opens a window summarizing the data represented in that cell. At the top of the summary are filters that allow users to refine their search results by attributes of the assay results or by vocabulary annotations to the genes analyzed. Users can download or forward results for further analysis using the export functions provided in the Genes and Assay results tabs.

The profile search offers two modes: Classical Expression Assays and RNA-Seq. Each mode is optimized for its respective data type. As a result, the “not detected” search differs in each mode. In Classical Expression Assay mode, the returned genes are either those recorded as not detected in the database (i.e. absent) or those for which expression in the specified anatomical structure and/or developmental stage is not recorded in the database (i.e. not analyzed). This accommodation reflects the tendency of authors to underreport the absence of gene expression when discussing classical expression results. For instance, when doing *in situ* studies, to be certain expression is truly absent the researcher would need to serially section the entire anatomical structure. As a result, it is easier to discuss where expression is present rather than where it is lacking. The Tissue × Gene Matrix tab, the default view of the Gene Expression Search Summary in Classical Expression Assay mode, allows users to distinguish the not detected and not analyzed cases (Fig. 3).

**Figure 4.**
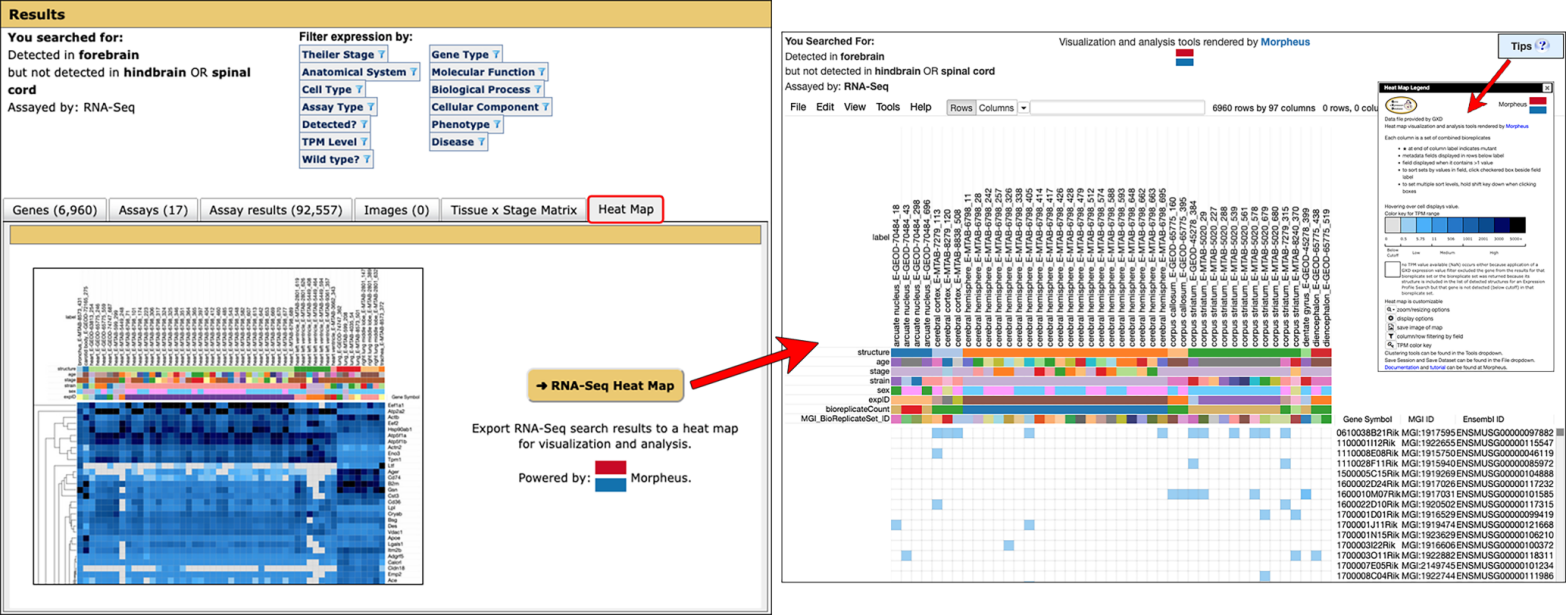
Heat Map tab of the Gene Expression Search Summary. When using the RNA-Seq mode of the Expression Profile Search, the default tab is the Heat Map (boxed in red in left image). The tab displays a static image of a generic heat map, but clicking on the gold → RNA-Seq Heat Map button will launch a browser window displaying the user’s returned data in Morpheus (right image). Developed by the Broad Institute, Morpheus is a powerful heat map visualization and analysis tool that offers a myriad of utilities for further display and analysis, including sorting and filtering capabilities, hierarchical clustering, nearest neighbor analysis, and visual enrichment features. GXD-curated metadata annotations, such as anatomical structure, age, developmental stage, strain, sex, and bioreplicate count (indicating the number of bioreplicate samples from which TPM values were combined during GXD data processing), can be used for sorting and clustering purposes. A Tips box (right image inset) provides guidance on interpreting the heat map and using Morpheus effectively. Additional information is provided in Baldarelli *et al.* [9].

RNA-Seq experiments, in contrast, have comprehensive genome coverage. Therefore, absence of expression information is complete for the anatomical structures studied. Accordingly, in RNA-Seq mode, the “not detected” search only returns genes below the TPM cutoff (i.e. absent). Please note the Expression Profile Search does not compare gene expression levels between samples/specimens, it only compares presence or absence of expression in samples/specimens annotated to the anatomical structures and/or Theiler stages selected. Thus, in RNA-Seq mode, TPM values are not considered in the search, except with respect to the TPM cutoff used to define whether expression is considered present or absent. As detailed below, the new Heat Map tab is the default view of the Gene Expression Search Summary for RNA-Seq data returned from the Profile Search (Fig. 4).

### New Cell Ontology Browser

**Figure 5.**
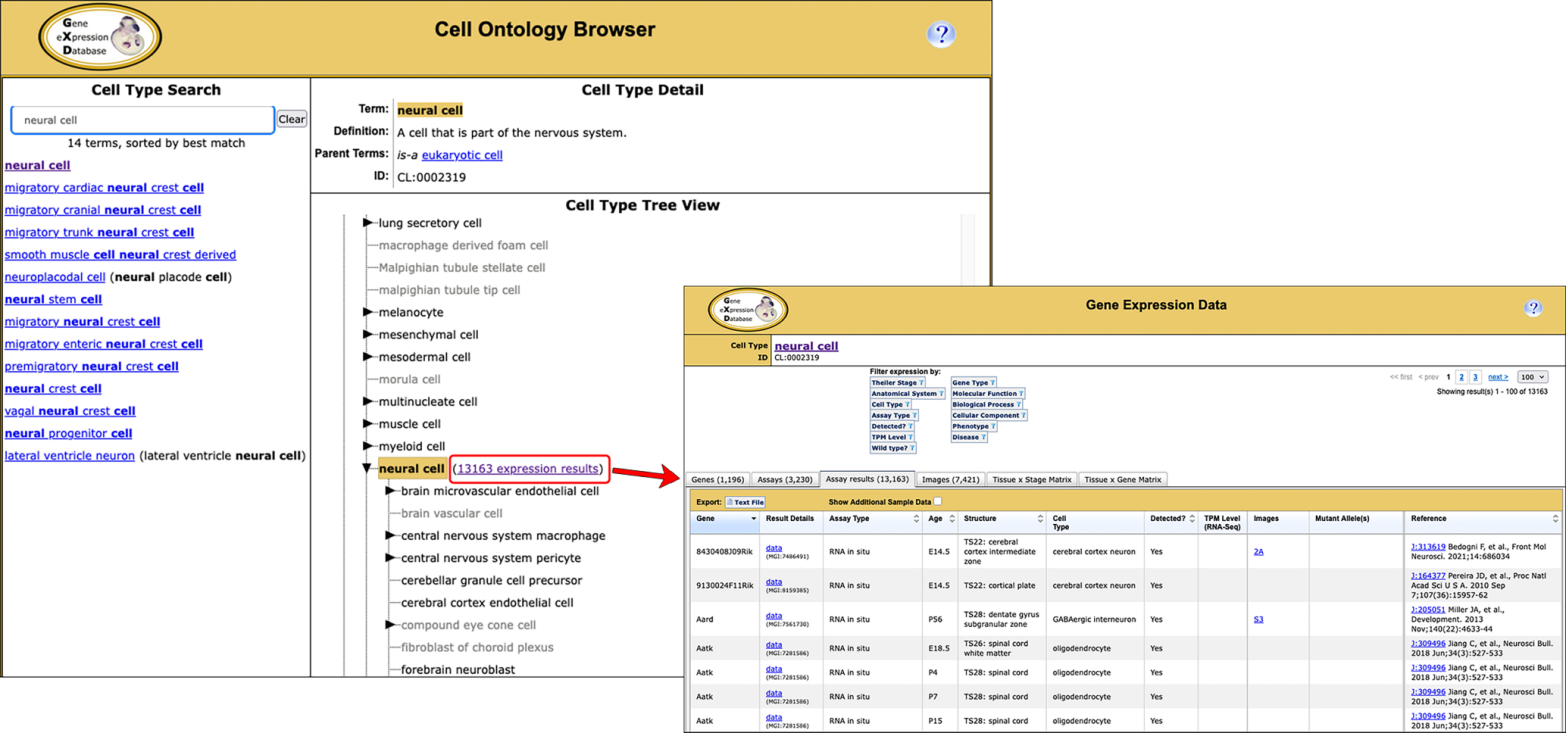
Using the Cell Ontology Browser. Users can search for a CL term by entering a text string or a CL accession ID into the Cell Type Search box (left image). Typing in the search box activates an autocomplete list displaying matching cell type terms and synonyms. When a term is selected, the Cell Type Detail and Tree View sections are updated, and the selected term is highlighted in gold. The Tree View displays the cell type term in the context of the classification hierarchy. It can also be used to browse for terms by expanding and collapsing branches. Greyed-out terms have no associated expression annotations; some of these cell types are not present in the mouse. The number of expression results associated with the selected cell type is displayed in the Tree View (boxed in red in left image). Clicking the link will lead to a summary page of expression results annotated to that term or one of its subterms (right image). Links in the summary provide access to the detailed expression records, such as the one shown in Fig. 1. More detailed instructions on how to use the Cell Ontology Browser are provided at https://www.informatics.jax.org/faq/GXD_cell_type.shtml.

The CL is a hierarchically structured, controlled vocabulary that describes a broad range of canonical biological cell types [18]. The CL is not organism-specific, encompassing cell types from prokaryotes to mammals, although its primary focus is on vertebrate cell types. To enable exploration of this ontology and to facilitate retrieval of expression data annotated to its terms, we have developed the Cell Ontology Browser (https://www.informatics.jax.org/vocab/cell_ontology). This tool can be used to search for and view descriptions of cell types, to explore the ontology hierarchy and locate specific terms in context, and to retrieve expression data associated with selected terms and their subterms (Fig. 5). Work is currently underway to add links to retrieve RNA-Seq and microarray experiments in our metadata index that analyze cell types.

### New tab and filter added to Gene Expression Search Summaries

GXD expression data searches yield a search summary page with tabbed data views (Figs. 3 –5). Each tab presents the search results at different levels of detail, and users can easily switch between the views. For searches that return RNA-Seq data, we have added a Heat Map tab (Fig. 4). It is the default tab for the Expression Profile Search RNA-Seq mode. While we provided a link to integrated Morpheus heat maps from the GXD Summary Page before, this new tab offers greater access to Morpheus, a heat map visualization and analysis tool created at the Broad Institute, which we have embedded into the GXD interface. This provides users with additional ways to display and analyze the RNA-Seq search results.

GXD search summaries feature filters that allow users to refine search results based on attributes of the assay results or by curated annotations to the genes analyzed (Figs 3–5). These enable users to take advantage of the comprehensive genetic, functional, phenotypic, and disease-related information in MGI. To further enhance this functionality, we have developed a new cell type filter that utilizes a set of high-level CL terms as organizational categories, allowing users to narrow search results to those annotated to cell type terms in those categories. This filter, along with others, is also available on the Genome Features and Vocabulary Terms tabs of the Quick Search results summary. The Quick Search can be found in the upper right corner of all MGI pages.

## User support

GXD provides support to its users through its staff, detailed online documentation, and quick tutorials. To get help, users can email mgi-help@jax.org or click the “Contact Us” link located in the navigation bar at the top of all web pages. The online documentation can be accessed by clicking on the question mark icon in the upper corner of most pages. Quick tutorials and other informational material can be found on the Help & Training tab of the GXD home page (https://www.informatics.jax.org/expression.shtml).

## Citing GXD

When citing data downloaded from GXD, please use the following format: These data were retrieved from the Gene Expression Database (GXD), Mouse Genome Informatics, The Jackson Laboratory, Bar Harbor, Maine, USA (https://www.informatics.jax.org) on [date (month, year) when you retrieved the data cited]. To cite the GXD database itself, please use this article.

## Data Availability

GXD is freely available at https://www.informatics.jax.org/expression.shtml.
